# Optimizing spatial allocation of seasonal influenza vaccine under temporal constraints

**DOI:** 10.1371/journal.pcbi.1007111

**Published:** 2019-09-16

**Authors:** Srinivasan Venkatramanan, Jiangzhuo Chen, Arindam Fadikar, Sandeep Gupta, Dave Higdon, Bryan Lewis, Madhav Marathe, Henning Mortveit, Anil Vullikanti

**Affiliations:** 1 Biocomplexity Institute & Initiative, University of Virginia, Charlottesville, Virginia, United States of America; 2 Department of Statistics, Virginia Tech, Blacksburg, Virginia, United States of America; 3 Fralin Life Science Institute, Virginia Tech, Blacksburg, Virginia, United States of America; 4 Biocomplexity Institute, Virginia Tech, Blacksburg, Virginia, United States of America; 5 Department of Computer Science, University of Virginia, Charlottesville, Virginia, United States of America; 6 Department of Engineering Systems and Environment, University of Virginia, Charlottesville, Virginia, United States of America; National Institutes of Health, UNITED STATES

## Abstract

Prophylactic interventions such as vaccine allocation are some of the most effective public health policy planning tools. The supply of vaccines, however, is limited and an important challenge is to optimally allocate the vaccines to minimize epidemic impact. This resource allocation question (which we refer to as VaccIntDesign) has multiple dimensions: when, where, to whom, etc. Most of the existing literature in this topic deals with the latter (to whom), proposing policies that prioritize individuals by age and disease risk. However, since seasonal influenza spread has a typical spatial trend, and due to the temporal constraints enforced by the availability schedule, the when and where problems become equally, if not more, relevant. In this paper, we study the VaccIntDesign problem in the context of seasonal influenza spread in the United States. We develop a national scale metapopulation model for influenza that integrates both short and long distance human mobility, along with realistic data on vaccine uptake. We also design GreedyAlloc, a greedy algorithm for allocating the vaccine supply at the state level under temporal constraints and show that such a strategy improves over the current baseline of pro-rata allocation, and the improvement is more pronounced for higher vaccine efficacy and moderate flu season intensity. Further, the resulting strategy resembles a ring vaccination applied spatiallyacross the US.

## Author summary

Annual vaccination campaigns continue to be one of the prime measures which help alleviate the burden of seasonal influenza. Due to production and logistic constraints, there is a need for prioritization policies associated with vaccine deployment. While there is general consensus on age-based or risk-based prioritization, spatial optimization of vaccine allocation has not yet been explored in sufficient detail. In order to do this, we develop a mechanistic model of influenza spread across the United States, and propose a greedy mechanism for spatial optimization. We test the methodology on different realistic scenarios with temporal constraints on vaccine production.

## Introduction

Infectious diseases are the largest cause of human mortality worldwide, leading to over 13 million deaths a year [1]. Respiratory diseases alone account for a large fraction of these infections—CDC reports that the buden of illness during 2017-18 influenza season was high in the United States, with an estimated 48.8 million illnesses and 959,000 hospitalizations [33], higher than any season since the 2009 pandemic. Therefore, controlling the spread of infectious diseases, especially influenza, remains an important priority for local, state, and federal governments in the US and countries worldwide. Pharmaceutical interventions (PI) such as the use of prophylactic vaccinations and anti-viral drugs remain one of the most effective methods for controlling the spread of infectious diseases, e.g., [2], [3]. However, these interventions are constrained by limited resource supply and the high logistics cost of delivering them over a large geographical region. These limitations have been an actively studied topic in public health policy research. The primary objective here is to devise allocation and distribution strategies to improve the overall effectiveness of the limited supply of vaccines. In this paper, we denote this problem as VaccIntDesign. Most of the existing literature has focused on identifying whom to allocate the vaccines to, based on age and disease risk [5] [6]. However, it is widely understood that seasonal influenza exhibits typical spatial trends [7]. Further, the vaccine allocation problem is temporally constrained by the production and availability schedule, while most prior studies on epidemic interventions have primarily focused on static allocation before the start of the epidemic [2]. Thus the when and where aspects of allocation become equally, if not more, relevant. In [8], the authors studied vaccine allocation and distribution during the 2009 H1N1 pandemic and found that for many states, the vaccines arrived far too late to be useful. While the authors define possible alternatives for the pro-rata vaccine distribution using axiomatic Operations Research (OR) models, in this paper, we adopt a simulation optimization approach, by using a mechanistic model of influenza spread across the US.

Solving VaccIntDesign is challenging due to multiple related factors. Epidemic spread is a very complex phenomenon, and is typically modeled by the SEIR class of non-linear models. Human contacts and mobility play a crucial role in the dynamics of epidemic spread [4] [9]. Therefore, solving VaccIntDesign requires a combination of the modeling of human mobility and epidemic spread, and designing a strategy for optimizing the allocation of vaccines subject to availability constraints.

### Related work

There is an abundance of literature on the modeling, analysis, and control of epidemics. We briefly mention three areas that are closely related to our paper, namely, mobility modeling, disease modeling, and designing interventions to control the spread of epidemics. We refer to [11] [12] for surveys on these topics.

#### Modeling social contact networks and human mobility

There is very limited data on social contact networks and human mobility, and so there has been a lot of work on developing realistic models using different kinds of datasets. Eubank et al. [4] developed a first principles based approach for constructing a realistic synthetic population by integrating over a dozen public and commercial datasets. Coarser models for some countries have been constructed using census and Landscan data, e.g., [13] [15]. However, none of these approaches considers long-distance travel outside an urban region. One of the earliest approaches for considering such travel was by Colizza et al. [9], who use information from airline data to construct a network-based representation of cities across the globe. However, airline flow does not account for all of spatial mobility, especially within the US. In [10], the authors construct a radiation model to predict commuter flows in the United States using data on road networks. Especially in the context of national scale disease spread, it is essential to have a model that combines both short-range and long-range human mobility in the United States.

#### Disease modeling

There are a number of variants of the SEIR type of models for disease spread, and their applicability depends on the specific assumptions that hold. The most commonly used models are compartmental in nature, assuming well-mixed populations within each compartment. A number of variants have been proposed, including stochastic models, multiple compartments to represent various subpopulations, branching processes, chain-binomial models, etc. Colizza et al. [9] use a patch model of the form we study in this paper. They study the role of long distance travel in the spread of epidemics, and use it to explain the SARS outbreak and to forecast other outbreaks. A different approach that is more suitable for heterogeneous populations is based on a network abstraction [4]. A lot of data is needed for developing such network-based models, and such models are usually computationally more intensive.

#### Designing interventions

Most compartmental models that have been used for studying optimal interventions are relatively simple, and can be solved using simple black-box optimization methods. An example is the work of Medlock et al. [2], who consider the problem of designing an optimal vaccine allocation for the 2009 H1N1 outbreak. They use an age-based coupled ODE model, and observe that the optimal solution is different from the CDC recommendation at that time. Similar studies have been done for other outbreaks, e.g., [16], who observe that prioritization of high-risk individuals leads to more effective strategies. However, most studies for designing vaccination policies do not take into account the spatiotemporal spread dynamics of seasonal influenza, nor the temporal constraints in vaccine production schedule. Thus there is need for coupling a mechanistic model of national-scale influenza spread with realistic vaccine uptake information for deriving an effective vaccine allocation algorithm.

### Our results

We develop a framework for national seasonal/pandemic influenza planning using realistic datasets, a mechanistic model of disease spread, and a greedy optimization algorithm for vaccine allocation. Our specific contributions are discussed below.

#### National-scale influenza model

We develop a national-scale metapopulation model for the spread of influenza by integrating both local and long-distance travel within the United States. We combine data on commuter mobility from the American Community Survey (ACS) with domestic airline passenger data from the Bureau of Transportation Statistics (BTS) to capture human mobility across the country. Next, we adopt a metapopulation approach to simulate epidemic spread at the spatial resolution of counties (patches) wherein the temporal travel matrix is used to represent the flow of people between these patches. While similar models exist in the literature [17], this is one of the first models to integrate realistic datasets on vaccine uptake, allowing us to address the VaccIntDesign problem.

#### Multi-stage Spatio-temporal calibration

Challenges in calibrating such a complex model include: (a) model non-linearity, (b) large number of parameters, (c) multiple spatio-temporal characteristics to calibrate to (regional attack size, peak timing, etc.), (d) lack of standardized and accurate infection counts. We use two-stage posterior exploration via importance sampling to calibrate the disease model to the total attack size (number of infections) and peak timings of the ten HHS (Health and Human Services) Regions. While peak timings for HHS regions can be obtained from the CDC ILINet (Influenza-like Illness Outpatient Surveillance Network) data, we use a novel approach to estimate total attack size for each region, and demonstrate the calibration performance for 2014-15 influenza season.

#### Vaccine allocation optimization

We develop a heuristic for finding a spatio-temporal vaccine allocation using a greedy strategy GreedyAlloc with a lookahead parameter, under a fixed vaccine availability schedule. We evaluate the algorithm’s performance by comparing it against a population proportional allocation strategy (baseline). Some of our key findings are: (a) For the scenario under consideration, GreedyAlloc leads to considerable reduction in the total attack size compared to the baseline; (b) the performance of GreedyAlloc improves with vaccine efficacy, and for influenza seasons with moderate intensity; (c) the identified vaccine allocation strategy resembles a ring-vaccination, applied spatially for the state-level adjacency matrix of the US.

**Fig 1:**
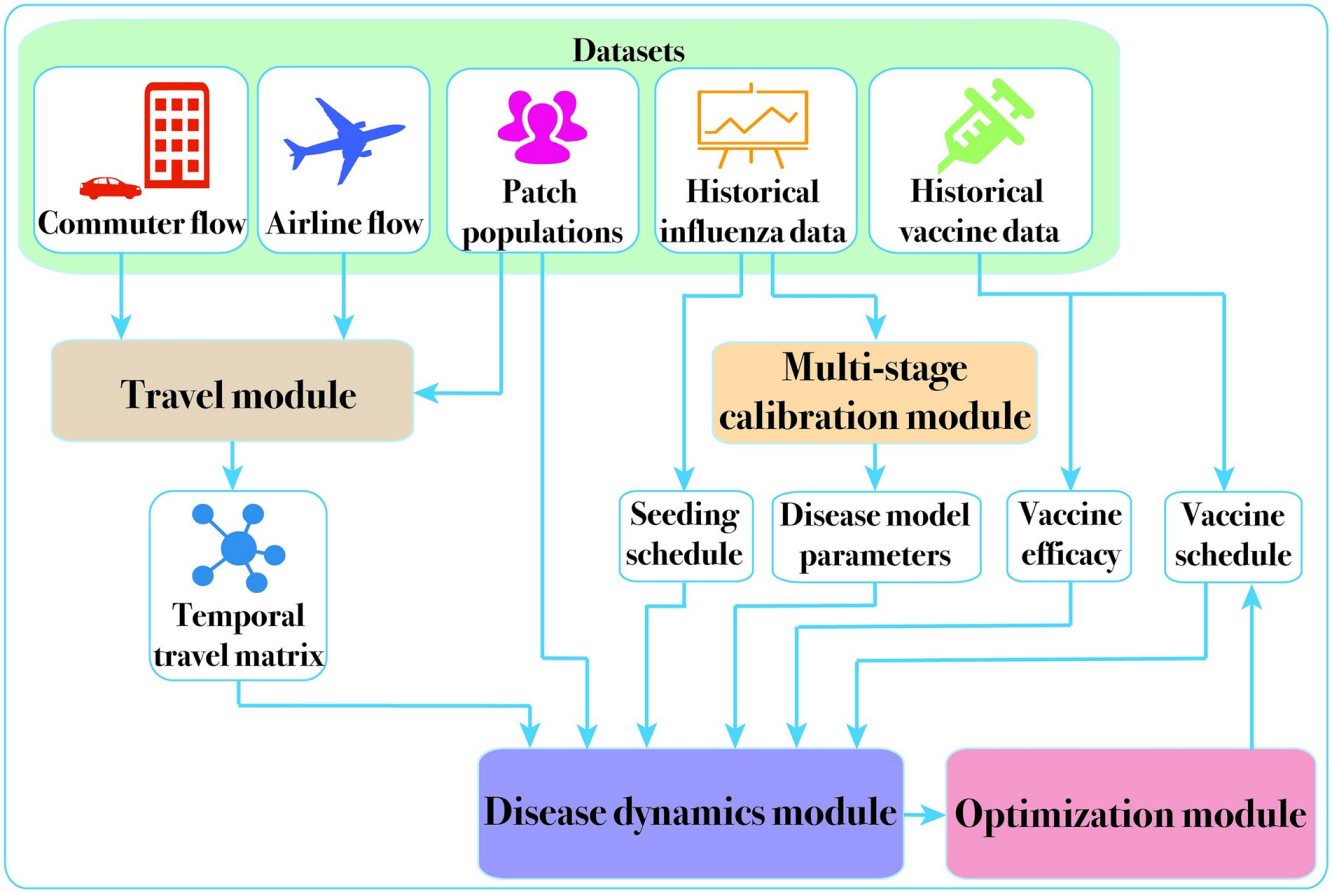
System diagram. Datasets used in the study are listed on top, and the four main modules of our framework are highlighted in the system diagram. The Travel Module uses long distance airline travel data and short-range commuter flow data to construct the county to county temporal travel matrix. This is used by the Disease Dynamics Module for simulating national scale epidemic spread via the metapopulation model. Additional inputs to this module include the seeding and vaccination schedule, disease model parameters, and vaccine efficacy. While some of these are fixed based on the study design, the Calibration Module uses historical incidence data to estimate the remaining disease model parameters. The calibrated disease model is then used as an oracle to compute the optimized vaccine allocation in the Optimization Module.

The overall framework that implements the steps above is shown in Fig 1. The modular approach allows the refinement of each module, when novel datasets, algorithms, and modeling techniques become available. In the Materials and Methods section, we briefly describe the key ideas involved in construction of the national influenza model, the calibration procedure and the vaccine allocation algorithm. We refer the reader to the S1 Appendix. Additional methods for more details on each of these modules. We then present the results of calibration and vaccine allocation using 2014-15 season data as a running example. A preliminary version of the model and early results for VaccIntDesign were reported in [18], and the current paper includes improved calibration process and provides more realistic estimates for the effectiveness of vaccine allocation. We have also made the national simulation model code and associated data available online [29].

## Materials and methods

### National-scale influenza model

Our approach in building the national scale model involves two broad steps:

- Developing a model of national level mobility that yields estimates of the fractions $\Theta_{ij}^{t}$ moving from county *i* to county *j* on day *t*.
- Developing the metapopulation model that uses Θ and a realistic vaccine allocation schedule *X* to produce the spatiotemporal spread of influenza and in particular, to compute the national attack size *f*(*X*).

#### Travel module

**Fig 2:**
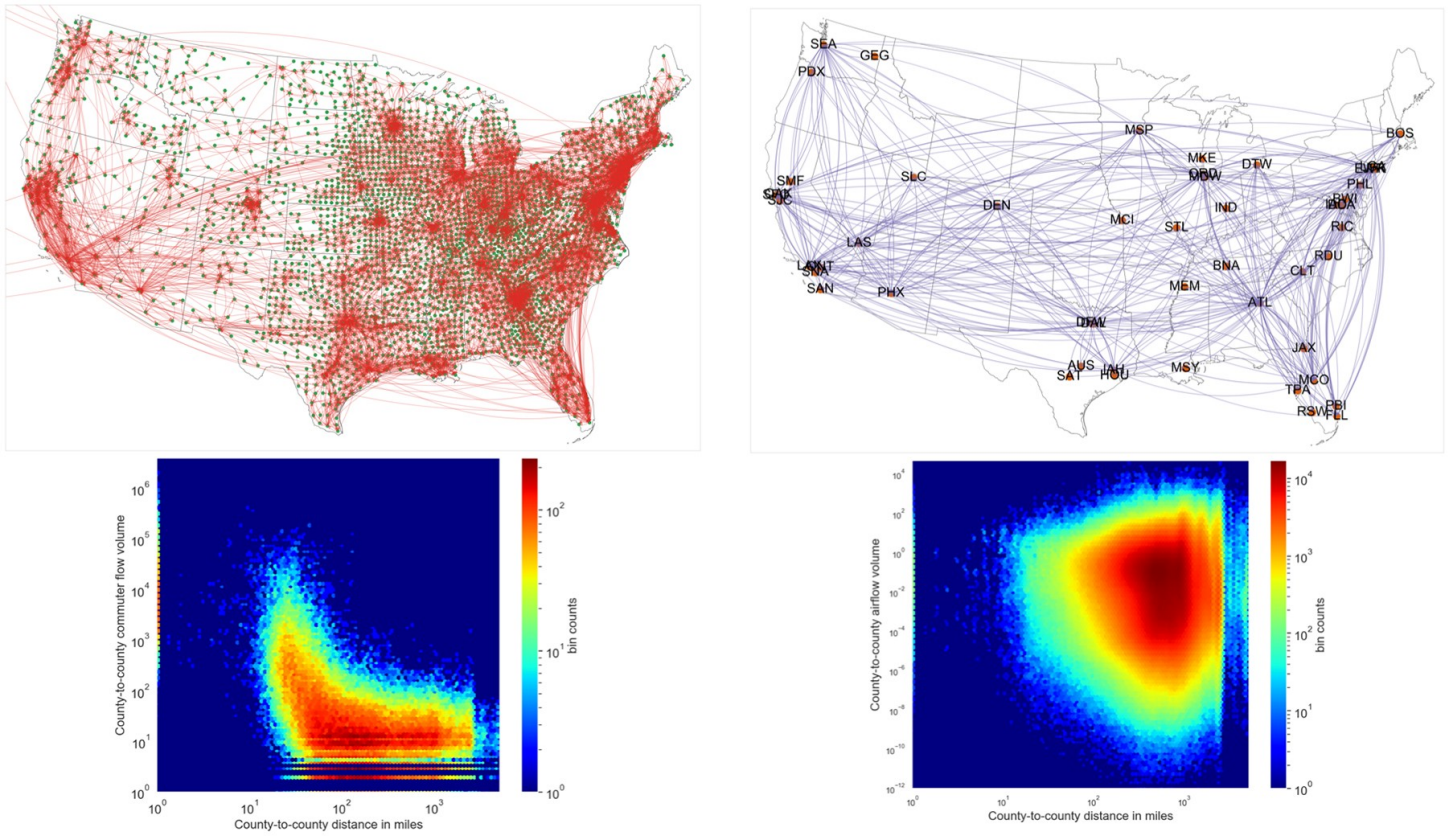
Travel datasets. Datasets used to capture short-range and long-range mobilities in the United States are depicted as networks on top (commuter data on the left, airline data on the right). Commuter data is shown between counties, whereas airline network is shown for key domestic airports in the United States for a chosen month (January). The distribution of flow volume with respect to pairwise county distances in shown as heatmaps for both the commuter and airline data. Airline flows are fractional because they are mapped to the counties that are served by them.

The purpose of the travel module is to generate the temporal travel matrix Θ representing flow of people between the patches (i.e., counties) on a daily basis. Each entry $\Theta_{ij}^{t}$ of the travel matrix represents the fraction of individuals in patch *i* spending their time in patch *j* on day *t*. The travel matrix Θ is synthesized using datasets pertaining to commuter flow and domestic airline traffic. These datasets are visualized in Fig 2.

#### Commuter data

The commuter flow dataset is obtained through the American Community Survey (ACS) [19] that, among other things, surveys individuals for their county of residence and county of workplace. ACS then provides population adjusted estimates of number of commuters between any pair of counties for a typical day. Fig 2 shows relationship between number of commuters from a source county to a destination county, and the distance between these counties. Other than the high volume of self-loop flows, most of the flows are concentrated in the 10–100 miles range.

#### Domestic airline data

Domestic airline flow data is obtained from the Bureau of Transportation Statistics (BTS) [20]. For a given year, this dataset provides the monthly total number of passengers who flew end-to-end between any two major airports. Fig 2 shows the number of airline passengers between counties, for a sample month. Contrasting this with that commuter flows, we find the large volume flows are concentrated in the 100–1000 miles range. In order to integrate this dataset with the county-level commuter mobility, we first identify the catchment area of each airport (defined as 120 miles surrounding the airport), and apportion the airport flows to counties based on their population in area of intersetion with the catchment area. We also distribute the monthly number of passengers uniformly through the month to get a daily count. Unlike a daily commuter, an airline passenger tends to stay longer in the visiting county. Therefore the effective number of airline passengers in the visiting county is obtained by scaling the flow by stay duration (in our model, we set the average stay duration as 3 days). More details on the travel network construction are provided in the S1 Appendix. Additional methods.

#### Disease dynamics module

Consider a population of individuals each of whom can be in one of the following states: Susceptible (S), Exposed (E), Infected (I), Recovered/Removed (R), Vaccinated (V). Compartmental models operate under a homogeneous mixing assumption, i.e., every individual can directly infect any other individual. Initially, the entire population is susceptible, except for a few initial infections possibly due to external contact. The disease progression is modeled by the evolution of number of individuals in each of the disease states, often described by a difference or differential equation.

Within each patch, we use an SEIRV model, and connect the patches using the travel matrix Θ^*t*^. When we extend the model for multiple patches, susceptible individuals can be infected by infectious individuals from other patches. This depends on the fraction of individuals moving from county *i* to county *j* on any given day, estimated using the temporal travel matrix Θ^*t*^.

The temporal travel matrix $\Theta_{ij}^{t}$ is used to calculate the force of infection among patches, without actually moving infected individuals around (i.e., virtual dispersal) [21]. We compute the conditional force of infection for an individual present in patch *j* by using the effective populations due to mobility. We can then calculate the unconditioned force of infection on a susceptible individual from patch *i*, as a component-wise product of (*a*) the probability of the individual being in a patch *j* ($\Theta_{ij}^{t}$) and (*b*) the conditional force of infection in patch *j* ($\beta_{j}^{\text{eff}}$). See S1 Appendix. Additional methods for the equations describing the model evolution, details on seeding and vaccination, and the sensitivity analysis of the model.

**Fig 3:**
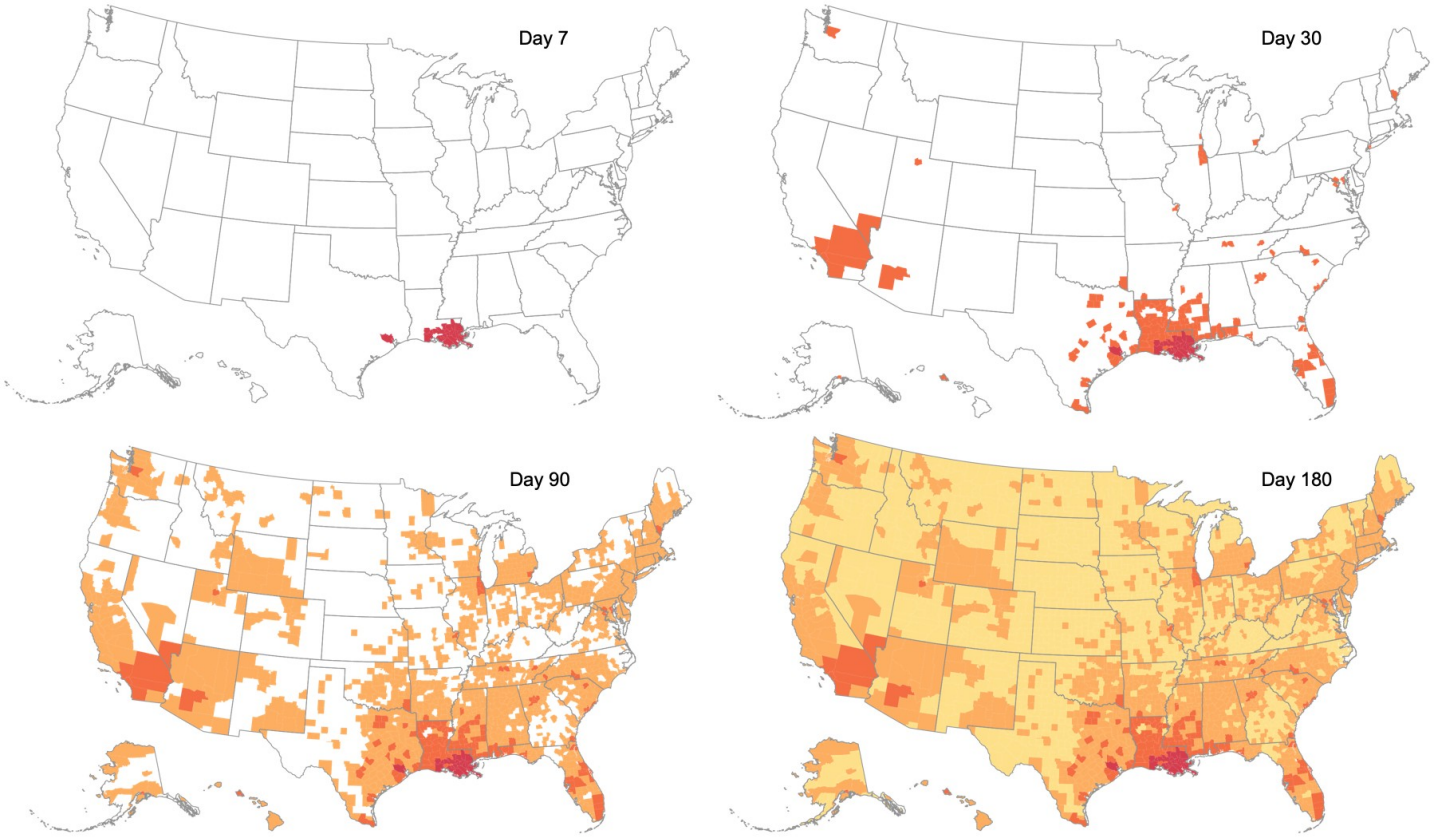
Model evolution. Spatio-temporal spread of influenza, for a sample scenario, seeded in Louisiana. Counties where the epidemic emerges by Day 7, 30, 90 or 180 are respectively shaded in red, orange, brown and yellow. We observe that the evolution of influenza spread exhibits both spatially local spread (aided by the commuter flow), and long range transmission events (aided by the domestic airline flow). This is especially evident in the transition between Day 7 to Day 30, where the epidemic originally seeded in southern counties of Louisiana spreads to other neighboring counties, as well as far away counties which have major airport cities (such as Seattle, Chicago, DC).

Note that the model is described through a system of difference equations, with each time step representing a day. The model is deterministic, and represents the average system trajectory. Fig 3 shows an example of the model evolution when simulated with the temporal travel matrix and a hypothetical seeding event in Louisiana.

### Spatio-temporal calibration

Model calibration is the process of estimating parameters of the computational model that can reproduce observed characteristics in the ground truth. In the context of epidemiology, beyond forecasting, calibrated models allow us to perform counterfactual (i.e., what-if) studies, and address resource allocation questions like VaccIntDesign. In this section, we will briefly describe our approach and the ground truth used for the two-stage calibration of the national-scale influenza model.

We begin with the assumption that the ground truth of interest *y* is a noisy version of the simulation model *η*(⋅) at some unknown input parameter configuration $\hat{\theta }$. We use a gaussian error model, which are simple and adopted widely for many applications, including epidemics [30, 31]. We adopt importance sampling [32] scheme to produce posterior realizations of the calibration parameters. We begin with sampling from an easy-to-sample importance distribution Im(*θ*) (say, uniform), and run the simulation model *η* at each of those samples. The importance weights are computed as the ratio of the posterior distribution (proportional to the product of likelihood and prior distribuion) and importance distribution evaluated for each of the samples. The samples along with the normalized weights then constitute an estimate of the posterior distribution.

Further, it is often useful to factorize the likelihood function, if possible, when the simulation model is required to be calibrated to several different criteria [31]. One possible way is to sequentially calibrate the model to different criteria. In addition to simplifying the computation of importance weights, the approach allows user to introduce more samples as needed using the intermediate calibrated parameter space. More details on the statistical framework and the two-stage posterior exploration is provided in S1 Appendix. Additional methods.

Finally, in our case, since we are interested in using a single calibrated model for the optimization study (as against a weighted ensemble provided by the posterior distribution), we consider the Maximum a Posteriori (MAP) estimate i.e., model configuration with highest frequency to be the calibrated model.

### Vaccine allocation optimization

We now consider the problem of determining the spatial allocation of vaccines across the US to minimize a chosen objective function. In addition to the complexity introduced by non-linear dynamics of the disease model, we also need to account for the temporal constraints imposed by vaccine production and delivery logistics. Formally, the VaccIntDesign problem involves determining the vaccine allocation vector *X* that minimizes the total attack size given by *f*(*X*). This can be expressed as:

$$\begin{array}{c} \begin{array}{cccc} & \underset{X}{\text{minimize}} & & f(X)\\ & \text{subject}\;\text{to} & & \sum_{i}X_{i,t}\leq B_{t},\;\;\text{for}\;\text{all}\;t, \end{array} \end{array}$$

where *B*_*t*_ is the total number of vaccines available at time *t*. Our goal in the VaccIntDesign problem is then determine the amount of vaccine allocated to each patch *i* at time *t*, denoted by *X*_*i*,*t*_.

- To reduce the dimensionality of the problem, we focus on allocating vaccines to the states, which are subsequently allocated to the counties proportional to their population. We will also consider the temporal allocation at the level of a weeks. Thus we will denote the allocation to state *s* ∈ *S* for week *w* ∈ *W* as *X*_*s*,*w*_ to improve readability.
- A possible objective function *f*(*X*) could be the total national attack size under vaccination schedule *X*, i.e., ∑_*i*_ *R*_*i*_(*T*), where *T* is the duration of the epidemic, and *R*_*i*_(*T*) denotes the number of individuals in Recovered state in patch *i* at time *T*.
- *B* is a *W*-dimensional vector, where *B*_*w*_ represents the number of vaccines available for week *w*.

The VaccIntDesign problem is very challenging, and its exact complexity remains open. A strategy that has been useful in many kinds of intervention design problems is to design a greedy allocation, which selects each decision variable based on the marginal improvement to the objective function. If the problem involves submodular maximization, such a strategy is guaranteed to give a constant factor approximation; see, e.g., [22] [23]. In contrast, VaccIntDesign involves a minimization, and the objective functions are neither submodular or supermodular, in general.

Nevertheless, the greedy strategy is a reasonable approach for designing vaccine allocation strategies, and we study it here with an allocation step size of *L*. The algorithm begins with an initial zero allocation. For each week *w*, the algorithm allocates the next set of *L* vaccines to the state *s* which leads to the maximum reduction in the objective value *f*(*X*). The algorithm is repeated for week *w*, until we exhaust *B*_*w*_, and then proceed to the next week’s supply of vaccines. Note that the computation of marginal benefit of allocating *L* additional vaccines to state *s* subject to population constraints, can be computed in parallel.

As a generalization, we have also included the lookahead duration *d* (in weeks) as an additional parameter. This means that the potential allocations at a greedy stage of week *w* are evaluated by their reduction of attack size at week min(*w* + *d*, *T*) where *T* is the total duration of the epidemic. While this includes the total attack size (full lookahead, when *d* ≥ *T*) as a special case, it also allows us to explore the resulting trade-off due to varying forecast horizons. The detailed algorithm is provided in S1 Appendix. Additional methods.

## Results

### Calibration performance

#### Calibration criteria

We calibrate the national influenza model at the level of Health and Human Services (HHS) regions. We use the timing of peak influenza activity across these regions to test the model’s ability to reproduce the spatiotemporal variation. To capture the spatial variation of impact, we use the total attack size in each HHS region. Both these calibration criteria are obtained from the Outpatient Influenza-like Illness Surveillance Network (ILINet) data provided by the CDC [34]. ILINet reports the weighted percentage of patient visits to healthcare providers for ILI each week and for each HHS region, wtILI(*h*, *w*).

**Fig 4:**
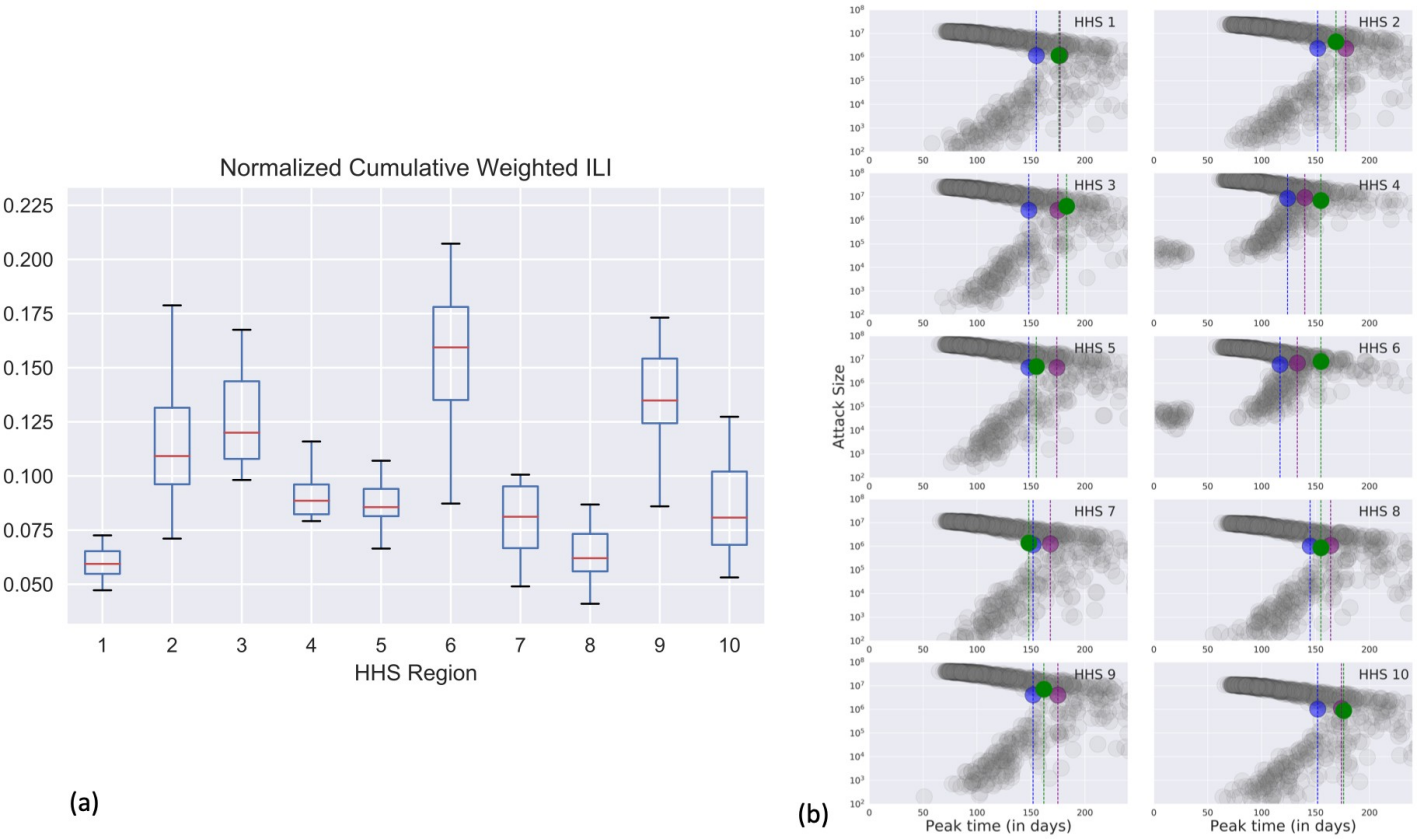
(a) Normalized cumulative weighted ILI% (norm_*h*_) for each HHS region, across past influenza seasons. (b) Results of multi-stage calibration. Target attack sizes for each HHS region and peak time are shown relative to the results of the calibration. For each HHS region, green circle represents the ground truth attack size, and the associated green dashed line represents the peak timing. In blue (purple) we show the targets achieved by best model chosen after first (second) stage of calibration. All combinations of attack sizes, peak timing achieved by the particles in the design space are shown in grey.

While peak timings are straightforward to obtain from ILINet data, regional attack sizes need to be inferred. We use CDC’s influenza burden estimates [33] with appropriate scaling (accounting for asymptomatic proportion) to obtain the national attack size AS_*N*_. We then use the normalized cumulative weighted ILI% of an HHS region (norm_*h*_) to proportionally obtain regional attack sizes.

$${\text{norm}}_{h}=\frac{\sum_{w\in W}\text{wtILI}\left(h,w\right)}{\sum_{\overset{h\in H}{w\in W}}\text{wtILI}\left(h,w\right)}$$

where *W* is the set of weeks of interest (typically from week 40 of a given year to week 20 of the following year, considered to be the influenza season) and *H* is the set of HHS regions. We observe that across seasons norm_*h*_ of HHS regions are quite consistent (as shown in the boxplots of Fig 4), and we used the median value across seasons. For our study, we used the peak timings of 2014-15 influenza season, and a national attack size of 40 million cases.

#### Calibrated parameters

The following is the list of simulation parameters (*θ*) in the national influenza model.

$$\begin{array}{ccc} 500\leq & s_{k}=\text{Number}\;\text{of}\;\text{initial}\;\text{seeds}\;\text{in}\;\text{county}\;k & \leq 2000,\;\;7\text{levels}\\ 0\leq & t_{k}=\text{Seeding}\;\text{timing}\;\text{in}\;\text{county}\;k & \leq 28,\;\;29\;\text{levels}\\ 0.4\leq & \beta =\text{Transmissibility} & \leq 0.9\\ 0.2\leq & vac_{\text{eff}}=\text{Vaccination}\;\text{efficacy} & \leq 0.6\\ 14\leq & vac_{\text{del}}=\text{Vaccination}\;\text{delay} & 28,\;\;15\;\text{levels}\\ 0\leq & A_{w}=\text{Stay}\;\text{duration} & \leq 5,\;\;6\;\text{levels} \end{array}$$ (1)

For seeding the influenza model, we used data from CDC ILI Activity Level Indicator [35] for the 2014-15 season. We chose to seed in Louisiana and Alabama, the two earliest states to reach High activity level in the season. Within these states, we chose to seed in the most populous counties (Lafourche parish, LA and Jefferson county, AL) respectively. The seeding policy is consistent with the finding that most influenza seasons likely start from the Southern US [24]. Given the seeding in two counties, we now have 8 parameters to be calibrated *θ* = (*s*_1_, *s*_2_, *t*_1_, *t*_2_, *β*, *vac*_eff_, *vac*_del_, *A*_*w*_). Note that the seeding time is time since the start of the simulation. We fix the remaining parameters of the disease model (such as *α*, *γ*) based on literature. In our study, *α* = 0.67, *γ* = 0.4 and correspond to mean incubation period of 1.5 days and mean infectious period of 2.5 days [14].

We begin with an initial set of *m* uniformly random parameter configurations according to (1). Based on initial runs, we choose to work with *m* = 1000. Full simulations are carried out at these 1000 configurations to obtain simulated regional attack sizes and peak timings. We then perform the two-stage calibration procedure described earlier, to obtain a MAP estimate of the best parameter configuration. For the gaussian likelihood (see S1 Appendix. Additional methods on Calibration methodology), we defined independent errors with a standard deviation of 20% around the calibration criteria [30]. While varying the standard deviation may affect the posterior distribution, it is to be noted that the MAP estimator remains unaffected.

After the first stage of calibration (i.e. regional attack sizes), the MAP estimate $\theta_{\left(1\right)}^{⋆}=\left(1000,1250,3,5,0.43,0.53,23,2\right)$. After the second stage of calibration (i.e.,regional peak timing), we get $\theta_{\left(2\right)}^{⋆}=\left(2000,1500,18,21,0.59,0.56,22,2\right)$. Fig 4 shows the results of multi-stage calibration, with the green, blue and purple circles respectively showing the calibration criteria, simulated output with $\theta_{\left(1\right)}^{⋆}$ and simulated output with $\theta_{\left(2\right)}^{⋆}$. We use $\theta_{\left(2\right)}^{⋆}$ as the calibrated parameter for the vaccine allocation study. We note that we are able to calibrate within 14% of the national attack size. The calibrated model is also within 24% on average for HHS region level attack sizes and 1.5 weeks (11 days) for the respective peak timings.

### Optimization study scenarios

For the current study, we begin with the disease model calibrated to the 2014-15 influenza season. Given the best fit model *M*_*θ*^⋆^_, we define the optimization study scenarios as follows: A scenario is defined by a (*v*, *E*) tuple and is derived by setting the vaccination efficacy to *v* in model *M*_*θ*^⋆^_ and calibrating the transmissibility *β* to achieve national attack size of *E* under pro rata vaccine allocation.

We do this to simulate multiple seasons that spread spatially like the 2014-15 season, but vary in their severity (captured by the national attack size *E*) and the efficacy of seasonal vaccine (captured by *v*). In our study setting, we construct 12 scenarios, where *v* takes values in {0.2, 0.35, 0.5} and *E* takes values in {40, 61, 73, 86} where the values are in millions of cases, corresponding to different severity levels based on past seasons of seasonal influenza. Thus for each target attack size *E*, we have three scenarios, in which *E* is achieved by assigning vaccines at *v* efficacy.

In our study (restricted to contiguous US, including DC), the number of states *S* = 49. Also, we set the number of weeks *W* = 40, roughly the period from September to May corresponding to the influenza season. Therefore, the allocation profile *X* has 1960 spatio-temporal dimensions. The temporal constraint *B* is based on historical vaccine uptake schedue available from CDC FluVaxView [36]. CDC FluVaxView provides monthly coverage estimates nationally for the past influenza seasons. We scaled it by the national population to get a vaccine uptake schedule and converted it to the temporal constraint *B*. Note that CDC also provides the vaccine supply and distribution schedule [37], however, we noticed a considerable delay between the supply and uptake schedules, so we chose to use the uptake schedule to reflect ground reality.

#### Effect of vaccine efficacy

**Fig 5:**
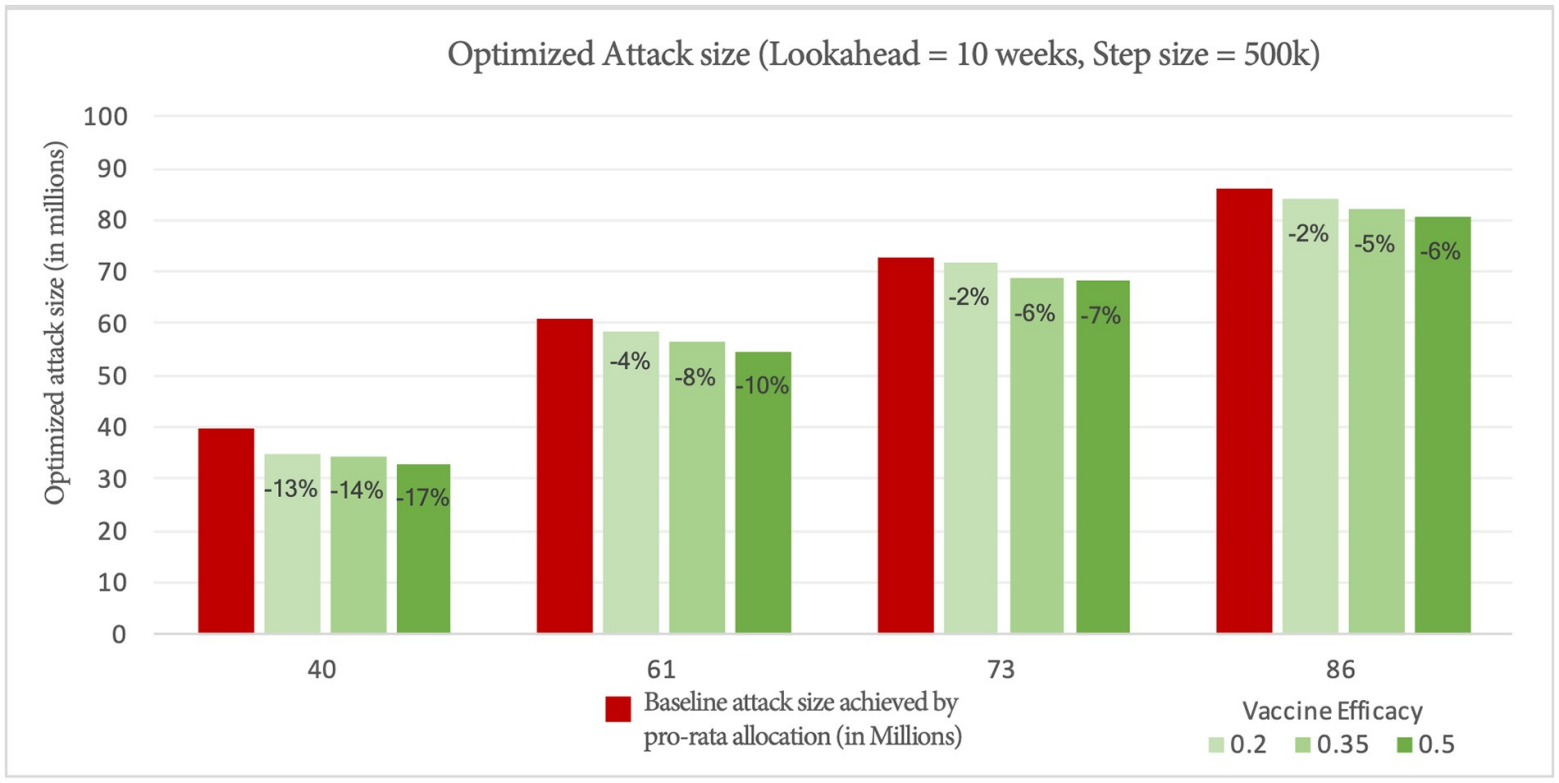
Episizes achieved by optimized allocation for different baseline episizes and vaccine efficacies.

Fig 5 shows the attack size under optimized allocation under a 10 week lookahead policy. In the least severe scenario (40 million cases), with the best vaccine efficacy (0.5), we are able to reduce the national attack size by up to 17%. On the other extreme, with 80 million cases and 0.2 vaccine efficacy, the allocation helps, but only minimally (2%). Another thing to note is that for a fixed attack size scenario, with increased vaccine efficacy, the benefit of optimized allocation increases. This makes sense, since this translates to more effective vaccines being moved around.

#### Spatio-temporal allocation of vaccines

**Fig 6:**
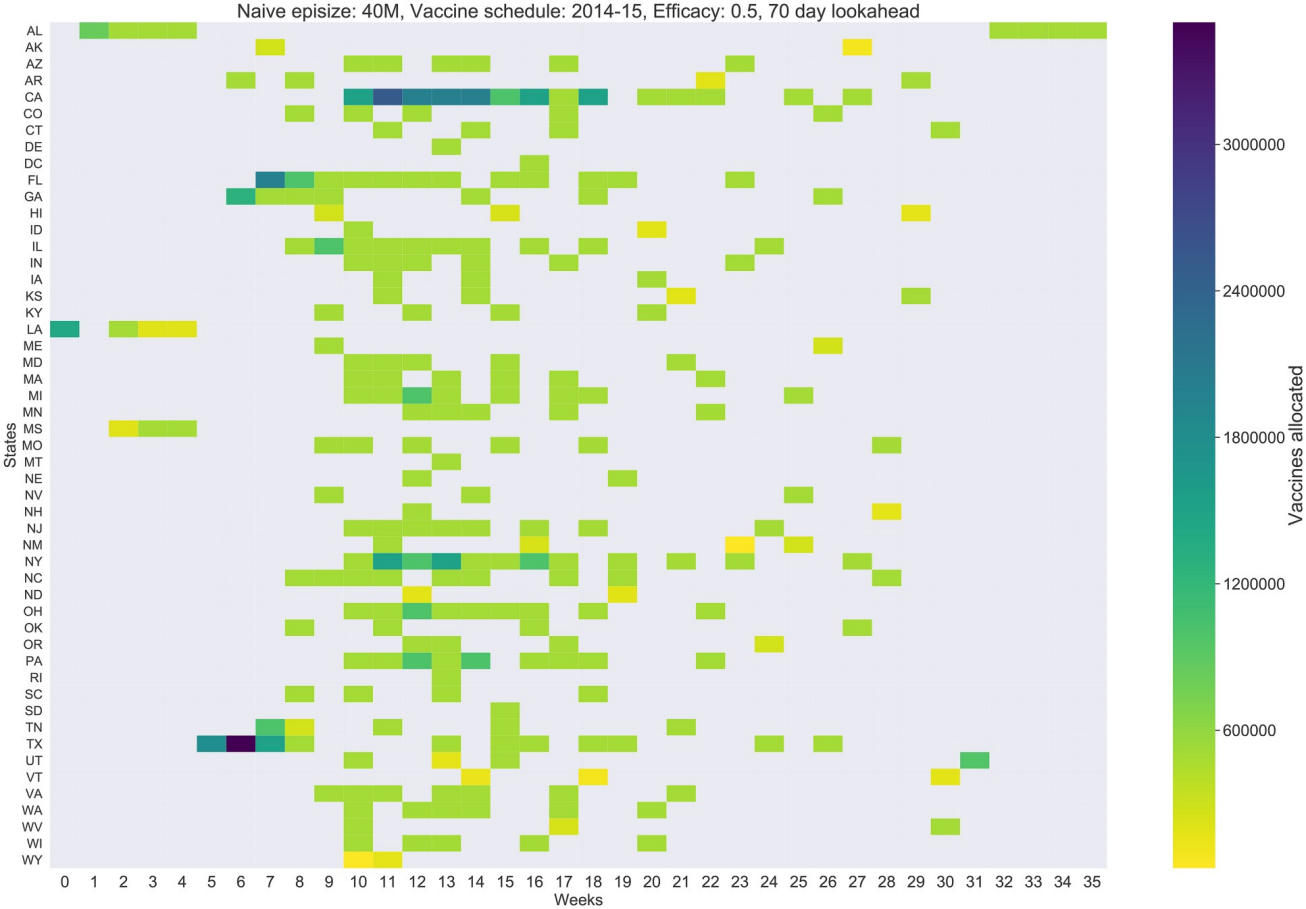
Weekly allocation of vaccines across states for the optimized allocation with 10 week lookahed, vaccine efficacy of 0.5 and baseline episize of 40M.

**Fig 7:**
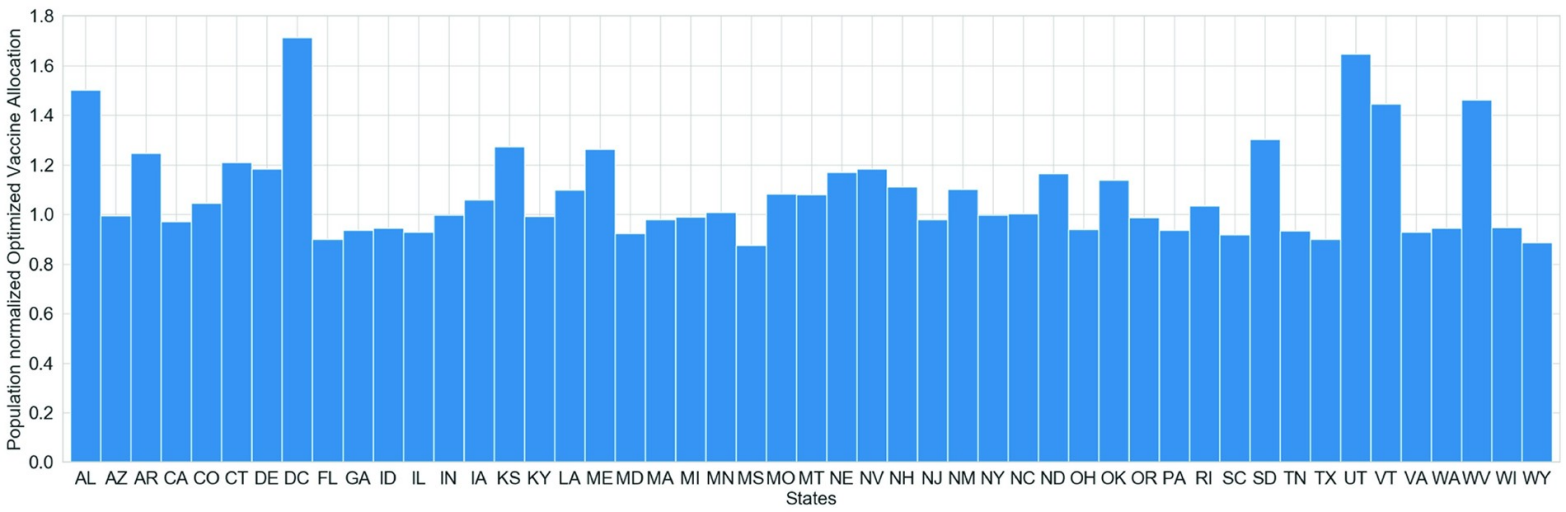
Population normalized total allocation of vaccines per state under the optimized schedule. A value of 1.0 indicates that the state received exactly the same amount of vaccines it would have received under the pro-rata schedule. Values greater (less) than 1.0 correspond to more (fewer) vaccines allocated under the optimized schedule than the pro-rata schedule.

Note that the algorithm outputs an allocation schedule, where for each week, each state is allocated *X*_*s*,*w*_ units of vaccines. This can be visualized using a 2-D heat-map as shown in Fig 6. It is useful to remember that all simulations are initialized in Louisiana and Alabama. Fig 6 shows the allocation by the 10 week lookahead policy with vaccine efficacy of 0.5. We observe that the optimized allocation begins allocating to Louisiana (state code LA), followed by its neighbors Alabama (AL), and Mississippi (MS). This is followed by larger states around Louisiana such as Texas, Georgia, and Florida. This is very different from a pro-rata allocation scheme (distributing the vaccines for each week proportional to state populations). This strategy also resembles a ring-vaccination (in social contact networks), applied spatially to regions around the origin of the epidemic. Further, even though we prioritize some of the states early in the season, the final vaccine allocation for each state is close to its pro-rata quota. As shown in Fig 7, most states receive their fair-share of vaccines.

#### Sensitivity to hyperparameters

**Fig 8:**
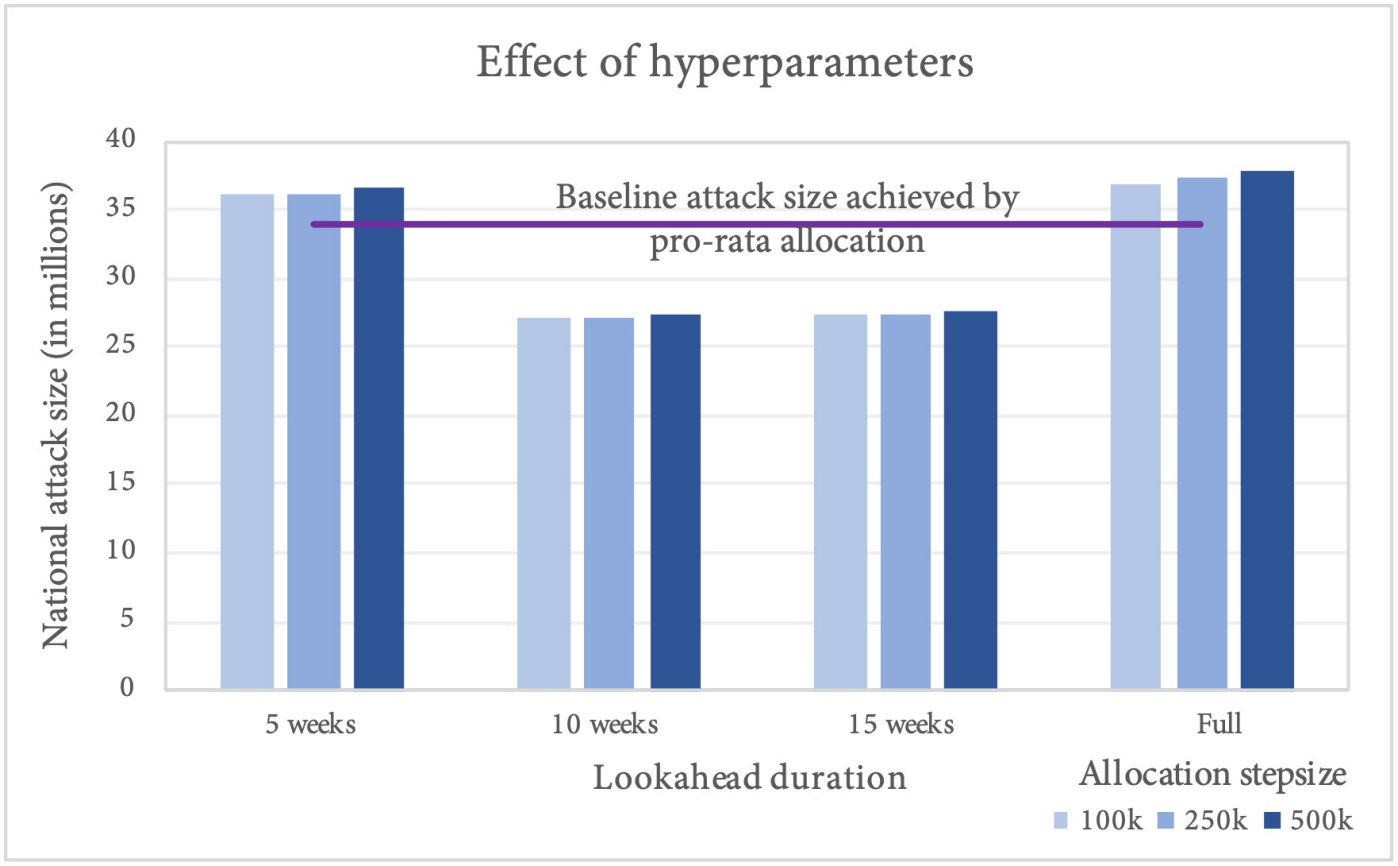
Effect of hyperparameters (lookahead duration and allocation stepsize) on algorithm performance.

We also tested the sensitivity of greedy algorithm performance to the hyperparameters of the algorithm, namely lookahead duration *d* and the vaccine allocation stepsize *L*. We tested the sensitivity on the best fit configuration for 2014, with total national attack size of 34 million. We observe from Fig 8 that the solution quality is pretty stable across allocation step size *L*, with marginal improvement for lower *L* (although the runtime for *L* = 100, 000 step size would be roughly 5 times as longer as that for *L* = 500, 000).

We also found that a medium lookahead policy (10 weeks) performed better than a short-term or full lookahead policy. This implies that allowing the algorithm to target the reduction of attack size a couple of months into the future, leads to improved performance in the final attack size. Short lookahead duration (5 weeks) does not perform well, because the lookahead is almost comparable to the delay for the vaccine to take effect. For longer lookahead, the algorithm allocates vaccines to states farther from the current epidemic activity to have the most impact on the total attack size at the end of the simulation. This, however, may not limit the spatial spread of the disease per se, and hence may be sub-optimal. As we can see, the medium lookahead policy, through its ring-vaccination style approach, ensures that the disease is curbed near its origin, thus leading to greater overall reduction in the attack size.

## Discussion

Current policies for vaccine interventions are designed based on a host of social and political issues, and tend to be fairly simplistic. For instance, Department of Health and Human Services (HHS) directives for targeting pandemic vaccines are based on age group [5], and the allocation of the national vaccine supply and other resources is typically done proportional to the state population. There has been a lot of interest in developing more effective interventions, e.g., [2] [4] [25] [26]. For instance, Medlock et al. [2] developed an optimal vaccination strategy for the H1N1 outbreak; their model showed a prioritization for a different age group than the ones recommended by CDC directives. All prior methods are restricted to simple models, and only focus on non-temporal interventions in which the allocation is done ahead of time. In reality, vaccine supply varies over time, and the real problem involves finding an allocation that respects the supply constraints and optimizes the epidemic outcomes.

Our current model can be extended in several ways. Firstly, the model calibration process can be refined to match more detailed trajectories of influenza spread, like the ILI % time series, or the in-season burden estimates being produced by CDC starting 2018-19 season [33]. Such approaches can then be used to do real-time forecasting and provide vaccine allocation recommendations for an ongoing influenza season. Further, instead of selecting the MAP model for vaccination study, one could use an ensemble of calibrated models based on the posterior distribution, thus being able to quantify uncertainty in the vaccine allocation policy’s effectiveness. Another aspect of the real-world dynamics currently not being captured in our model is that of residual immunity. The national influenza model can be improved by taking into account the co-circulating and dominant influenza strains, as well as the strains present in the recommended vaccine for the season. Note that while improving over pro-rata allocation, greedy algorithm, even with the lookahead duration, may lead to sub-optimal policies. One can develop algorithms that earmark resources for regions with high spreading capacity, thus potentially improving the effectiveness of vaccine allocation.

Finally, the logistics of the supply of medical resources, such as medicines, medical equipment (e.g., ventilators), and medical staff is also very complex. The health infrastructure is generally optimized for typical demand for such resources, and any surge, as would happen during a pandemic outbreak, would place a severe strain on hospitals. Ajao et al. [27] show that over 50,000 ventilators might be needed in the event of a national influenza pandemic outbreak. Since local and state health systems are usually unprepared for such a surge in demand, the Office of the Assistance Secretary for Preparedness and Response (ASPR) maintains a stockpile of mechanical ventilators in strategic locations [38], which can be deployed during an emergency. While existing efforts partially address the question of optimizing stockpile redistribution [28], a mechanistic model like the one developed in this paper will help design better national-scale studies for pandemic preparedness exercises, and develop strategies for allocation of vaccines and other resources during such emergencies.

In conclusion, we have presented a national level seasonal influenza model, based on short-range and long-range mobility datasets, and used it to optimize the spatio-temporal allocation of vaccines. For the scenario under consideration, we find that the national attack size can be reduced by up to 17% by allocating the early vaccines to regions around the origin of the epidemic. Most states still end up with close to their overall pro-rata quota of vaccines, however, these findings demonstrate that shifting when and where these vaccines are administered has a sizable impact on the national attack size. Achieving these optimal outcomes would require better surveillance and the ability to accelerate vaccine uptake at will, which presents multiple challenges. However, the study shows there is ample room for improvement and this framework provides means for developing a play-book for epidemic containment.

## Supporting information

S1 Appendix Additional methods. Additional technical details pertaining to the disease model, calibration framework and optimization algorithm are provided.
(PDF)
