# Supplementary material for: Optimizing spatial allocation of seasonal influenza vaccine under temporal constraints
Source: PLoS Comput Biol. 2019 Sep 16;15(9):e1007111. doi: 10.1371/journal.pcbi.1007111 (PMC6762205; doi:10.1371/journal.pcbi.1007111)
Supplement: S1 Appendix — Additional technical details pertaining to the disease model, calibration framework and optimization algorithm are provided. (PDF) [file pcbi.1007111.s001.pdf]

Optimizing spatial allocation of seasonal influenza vaccine under temporal constraints: Supplementary Material

September 11, 2019

Disease Model

Travel Module

Let P_i be the population of county i and C_{ij} the number of individuals with county of residence i and county of workplace j. Often $P_i - \sum_j C_{ij} > 0$, and reflects the non-commuting population in county i.

Let F^m_{uv} represent the number of monthly passengers from airport u to airport v in the month m. Let airport u's *catchment area* (120 miles surrounding the airport) share a non-empty intersection with county i, and let W_{iu} be the population residing in that intersection. Likewise, let W_{jv} be the population residing in the intersection of airport v's catchment area and county j. Assuming proportional distribution of airline passengers, we can derive the entries of county-county airline flow matrix as:

$$A^m_{ij} = \frac{1}{d_m} \frac{W_{iu}}{\sum_k W_{ku}} \times \frac{W_{jv}}{\sum_l W_{lv}} F^m_{uv}$$

where d_m is the number of days in month m. Note that A^m_{ij} then represents the number of airline traveler originating in county i and traveling to county j on a typical day of month m. In order to account for longer stays by airline travelers, we use Δ to denote their average stay duration.

On a given day of month m, the effective number of individuals from county i who are in county j is then given by $O^m_{ij} = C_{ij} + \Delta * A^m_{ij}$. The monthly aggregated travel matrix Θ^m_{ij} is obtained by normalizing as follows:

$$\Theta^m_{ij} = \begin{cases} \dfrac{O^m_{ii} + (P_i - \sum_j O^m_{ij})}{P_i} & \text{if } i = j \\ \dfrac{O^m_{ij}}{P_i} & \text{otherwise} \end{cases}$$

This ensures that Θ^m is row stochastic, i.e., $\sum_j \Theta^m_{ij} = 1$. Since we need a daily travel matrix, we will use $\Theta^t = \Theta^m$ for every day t in month m.

Disease Model

Single isolated patch Consider a population of N_i individuals in patch i. Let $S_i(t), E_i(t), I_i(t), R_i(t)$ denote the number of individuals in the Susceptible (S), Exposed (E), Infected (I), Recovered/Removed

(R) states respectively, at time t. The dynamics of epidemic spread in an *isolated* patch is described by the following set of equations.

$$\begin{aligned}
\Delta S_i(t) &= -\beta S_i(t)\frac{I_i(t)}{N_i} \\
\Delta E_i(t) &= \beta S_i(t)\frac{I_i(t)}{N_i} - \alpha E_i(t) \\
\Delta I_i(t) &= \alpha E_i(t) - \gamma I_i(t) \\
\Delta R_i(t) &= \gamma I_i(t)
\end{aligned}$$

where β denotes the transmissibility (which quantifies the rate at which an infected individual spreads the infection to each susceptible contact), and α and γ denote the incubation and recovery rate, respectively.

Extension for multiple patches In the metapopulation model, given the travel matrix Θ^t, let $I_j^{\texttt{eff}} := \sum_i \Theta_{ij} I_i$ and $N_j^{\texttt{eff}} := \sum_i \Theta_{ij} N_i$ be the effective number of infected individuals and effective population of patch j after travel on a given day. One can then write the conditional force of infection for an individual in patch j as

$$\beta_j^{\texttt{eff}} := \beta \frac{I_j^{\texttt{eff}}}{N_j^{\texttt{eff}}}$$

For county i, the evolution of $\mathcal{Z}_i = [S_i, E_i, I_i, R_i, V_i]$ from t to $t+1$ can be defined as follows:

$$\mathcal{Z}_i(t+1) = \mathcal{Z}_i(t) + \Delta \mathcal{Z}_i(t)$$

where,

$$\begin{aligned}
\Delta S_i &= -\Delta V_i - \sum_{j=1}^{K} \Theta_{ij} \beta_j^{\texttt{eff}} S_i \\
\Delta E_i &= \sum_{j=1}^{K} \Theta_{ij} \beta_j^{\texttt{eff}} S_i - \alpha E_i \\
\Delta I_i &= \alpha E_i - \gamma I_i \\
\Delta R_i &= \gamma I_i
\end{aligned}$$

and ΔV_i is the number of newly vaccinated nodes (at time t).

Seeding The epidemic is seeded in specific counties with a specified number of initial infectious cases. In these counties, the state vector Z_i is initialized to $[P_i - X_i, 0, X_i, 0, 0]$, where P_i is the population size of patch i, and X_i is the corresponding number of initial cases. For the remaining counties, the entire population is assumed to be susceptible, i.e, initialized to $[P_i, 0, 0, 0, 0]$.

Vaccinations Vaccinations are administered on a daily basis based on the vaccine schedule. ΔV_i^t is used to denote the number of individuals vaccinated in patch i on day t. The model also accounts for vaccination efficacy and a time delay for the vaccination to take effect.

Sensitivity Analysis

To understand the model's sensitivity to input parameters, we study the model response characteristics with respect to its inputs. We adopt the Gaussian Process-based sensitivity analysis framework (GPMSA) as described in [1, 2]. The basic remise of GPMSA is to represent the high dimensional model output with fewer basis vectors, and use a Gaussian Process model to emulate the basis coefficients. The resulting Gaussian Process model combined with the basis representation serves as a surrogate to the original simulation model.

The mathematical model is written as

$$\eta(\theta) = \sum_{i=1}^{p_u} K_i w_i(\theta) + \epsilon$$

where η represents the disease model output and θ is the vector of input parameters. K_i's are the bases obtained from m model runs, and each basis loading w_i is then modeled as zero mean Gaussian Process. We assume zero mean Gaussian for the noise term, ϵ. The number of bases, p_u, can be used to control the level of approximation to the original disease model, and K_i's are obtained using SVD. Covariance function of the gaussian process is defined so that there is exactly one parameter to be estimated for each input dimension to fully specify the GP model. Model fitting is done using bayesian updates as described in [1].

To begin with, we run the disease model at $m = 100$ different input locations, sampled according to a latin-hypercube based random design over d dimensional input parameter space. Here, we chose the following parameters as our input to the simulation: seed amount and seeding time for a few potential counties of origin, disease transmissibility, vaccine efficacy, vaccination delay, and stay duration (used in the travel module to combine commuter and airline flows), and attack size as the simulation response. These initial model runs serve as a training set for building a surrogate, which is then used to do a sensitivity analysis. Fig. A shows the sensitivity plots for the original disease model, by plotting the national attack size as we vary each of the parameters unilaterally over their range.

Calibration methodology

Statistical Methodology

Let y denote the quantity of interest (attack size, for instance) from the physical process and $\eta(\theta)$ denote the corresponding output from the simulation model at input parameter configuration θ. With the assumption that the ground truth is a noisy version of simulated output at some unknown input setting $\hat{\theta}$, i.e. $y = \eta(\hat{\theta}) + \epsilon$, given a prior distribution $\pi(\theta)$ for the true parameter vector θ, the resulting posterior distribution $\pi(\theta|y)$ for θ is given by

$$\pi(\theta|y) \propto L(y|\eta(\theta)) \cdot \pi(\theta), \qquad (1)$$

where the likelihood term $L(y|\eta(\theta))$ depends on the distribution of ϵ. Assuming an independent gaussian noise, the likelihood can be written as

$$L(y|\eta(\theta)) \propto \exp\left\{\frac{1}{2}(y - \eta(\theta))'\Sigma_y^{-1}(y - \eta(\theta))\right\},$$

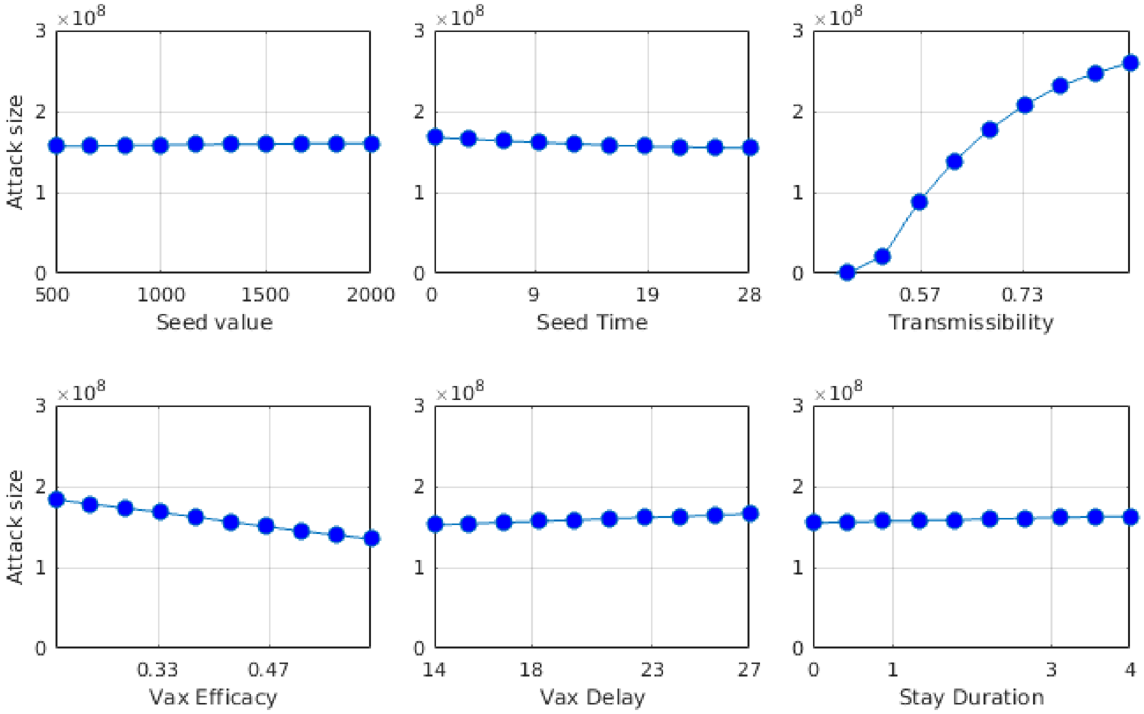

Figure A: **Model Sensitivity** Sensitivity of the model's output (in this case total number of infections, i.e., attack size) is shown with respect to the different calibration parameters. Note that the response curves are smooth, and except for transmissibility, the attack size is not very sensitive to the other parameters. Of the chosen parameters, one can note that seed time and vaccination efficacy have an inverse effect on attack size.

where, Σ_y is a diagonal matrix of order same as the length of y with diagonal entries being the corresponding variances.

Given the importance distribution $\text{Im}(\theta)$, the importance weight for a sample model configurations θ^* is then computed as:

$$w(\theta^*) = \frac{\pi(\theta^*|y)}{\text{Im}(\theta^*)} = \frac{L(y|\eta(\theta^*)) \cdot \pi(\theta^*)}{\text{Im}(\theta^*)} \tag{2}$$

The weights are normalized by $w(\theta^*) = \frac{w(\theta^*)}{\sum w(\theta^*)}$. A re-sample from the sample θ^*s according to the normalized weights $w(\theta^*)$ constitutes a sample from the distribution $\pi(\theta|y)$.

Two stage posterior exploration For illustration purposes, let us assume we want to calibrate our influenza model according to two different ground truth criteria $y = (y_1, y_2)$. Similarly, we

rewrite the model output as $\eta(\theta) = (\eta_1(\theta), \eta_2(\theta))$, and correspondingly the likelihood becomes:

$$\begin{aligned} L(y|\eta(\theta)) &= L_1(y_1|\eta_1(\theta)) \times L_2(y_2|\eta_2(\theta)) \\ &\propto \exp\left\{\frac{1}{2}(y_1 - \eta_1(\theta))'\Sigma_{y_1}^{-1}(y_1 - \eta_1(\theta))\right\} \times \\ &\quad \exp\left\{\frac{1}{2}(y_2 - \eta_2(\theta))'\Sigma_{y_2}^{-1}(y_2 - \eta_2(\theta))\right\} \end{aligned} \tag{3}$$

In the two stage set up, a partial calibration is done using an intermediate posterior $\pi(\theta|y_1) \propto L_1(y_1|\eta_1(\theta))\pi(\theta)$ based on y_1. At this step, the calibrated simulation should agree with the partial ground truth y_1. The next step is exactly similar as above, except a new prior same as the intermediate posterior $\pi(\theta|y_1)$, and the likelihood $L_2(y_2|\eta_2(\theta))$, to obtain the final posterior distribution,

$$\pi(\theta|(y_1, y_2)) \propto L_2(y_2|\eta_2(\theta))\pi(\theta|y_1).$$

Factoring the likelihood also aids in importance sampling in the following manner. To produce calibrated input parameters using the intermediate posterior $\pi(\theta|y_1)$, one can simply follow the steps in Eq. (2), and calculate the normalized importance weights $w_1(\theta^*)$, using an uniform importance distribution. For the next stage, we use $\pi(\theta|y_1)$ as the importance distribution. The un-normalized importance weights are just the second likelihood $L_2(y_2|\eta_2(\theta))$ evaluated for each θ from $\pi(\theta|y_1)$ (follows from Eq. (2)). Here, note that the samples obtained from the full posterior $\pi(\theta|(y_1, y_2))$ via two-stage procedure is the same as one would have gotten by directly sampling from it.

Vaccine optimization

Recall the VaccIntDesign problem which is formally defined as:

$$\begin{aligned} \underset{X}{\text{minimize}} \quad & f(X) \\ \text{subject to} \quad & \sum_i X_{i,t} \le B_t, \quad \text{for all } t, \end{aligned}$$

In our study, we focus on optimizing the weekly state-wise allocation $X_{s,w}$, subject to budget constraints $B_w, w \in W$. We also use the generalized objective function $f(X, T'_w)$ in each stage of the greedy algorithm, where $T'_w = \min(w + d, T)$ and d is the lookahead duration. The detailed greedy algorithm for vaccine allocation is given in Algorithm 1.

Running time. The `while` loop of the algorithm executes $\lceil \sum_w B_w/L \rceil$ iterations. Within each iteration, the algorithm makes S oracle calls for each state s. Therefore, the total number of calls to the disease simulation is $\lceil \sum_w B_w/L \rceil S$. Within each iteration of the `while` loop, the evaluation of marginal contributions $Z_{s,w}$ can be done in parallel, which reduces the running time to $\lceil \sum_w B_w/L \rceil$.

Algorithm 1 GreedyAlloc

```
X_{s,w}(0) = 0 for each s, w
u = 0
for w ∈ W do
    T'_w = min(w + d, T)
    while B_w > 0 do
        alloc = min(L, B_w)
        for s ∈ S do
            Z_s = -1
            if \sum_{w'=0}^{w} X_{s,w'} + alloc < P_s then
                \hat{X} = X(u)
                \hat{X}_{s,w} += alloc
                Z_s = f(X(u), T'_w) - f(\hat{X}, T'_w)
            end if
        end for
        s^{opt}(u) = argmax_s Z_s
        X(u + 1) = X(u)
        X_{s^{opt}(u),w}(u + 1) += alloc
        B_w -= alloc
        u += 1
    end while
end for
```

References

[1] Higdon D, Gattiker J, Williams B, Rightley M. Computer model calibration using high-dimensional output. Journal of the American Statistical Association. 2008;103(482):570–583.

[2] Gattiker J, Myers K, Williams B, Higdon D, Carzolio M, Hoegh A. Gaussian process-based sensitivity analysis and Bayesian model calibration with GPMSA. Handbook of Uncertainty Quantification. 2016; p. 1–41.

[3] Venkatramanan S, Chen J, Gupta S, Lewis B, Marathe M, Mortveit H, et al. Spatio-temporal optimization of seasonal vaccination using a metapopulation model of influenza. In: Healthcare Informatics (ICHI), 2017 IEEE International Conference on. IEEE; 2017. p. 134–143.
